# Exposure of *Apis mellifera* (Hymenoptera: Apidae) colonies to imidacloprid impairs larval development, promotes oxidative stress in pupae, and induces changes in the midgut of adult bees

**DOI:** 10.1186/s40659-024-00571-5

**Published:** 2025-01-21

**Authors:** Daiani Rodrigues Moreira, Tuan Henrique Smielevski de Souza, Douglas Galhardo, Cinthia Leão Figueira, Samara Calvi Baulli, Breno Gabriel da Silva, Francieli das Chagas, José Washington Santos Oliveira, Jean Samel Rocha, Angélica de Souza Khatlab, Eliane Gasparino, Vagner de Alencar Arnaut de Toledo, Adriana Aparecida Sinópolis Gigliolli, Maria Claudia Colla Ruvolo-Takasusuki

**Affiliations:** 1 Department of Biotechnology, Genetics and Cell Biology, State University of Maringá, Maringá, Paraná 87020-900 Brazil; 2 Department of Animal Science, State University of Maringá, Maringá, Paraná 87020-900 Brazil; 3 Department of Exact Sciences, Escola Superior de Agricultura “Luiz de Queiroz” - University of São Paulo, Piracicaba, São Paulo 13418-900 Brazil; 4 Centre of Molecular Biodiversity Research, Leibniz Institute for the Analysis of Biodiversity Change, Zoological Research Museum Alexander Koenig, Adenauerallee 160, 53113 Bonn, Germany

**Keywords:** Fat cells, Field exposure, Honey bee, Redox system

## Abstract

Bees are essential pollinators that contribute to maintaining biodiversity and increasing agricultural production. However, by foraging on agricultural crops, bees may become contaminated with compounds used for pest control. In this study, we exposed bee (*Apis mellifera* L.) colonies to the insecticide imidacloprid (IMD) under field conditions to assess the occurrence of oxidative stress in larvae and pupae and investigate morphological changes in the fat body and midgut of larvae and midgut of adult bees. The apiary area was divided into three groups: control, commercial formulation containing IMD (Evidence^®^ 700WG) (IMD_CF_), and IMD active ingredient (Sigma–Aldrich) (IMD_AI_). Treatment groups were fed syrup containing 1 µg L^−1^ IMD, whereas the control group was fed syrup only. Compared with the control, larvae exposed to IMD_CF_ or IMD_AI_ for 42 days exhibited morphological changes in the external body, midgut, and fat body. The midgut of adult bees contaminated with IMD_CF_ showed only structural remnants of the peritrophic membrane and absence of regenerative cell nests. Oxidative stress analyses revealed that IMD_CF_-exposed larvae had higher nitrite and carbonylated protein contents and lower catalase and superoxide dismutase activity than control individuals. In pupae, IMD_AI_ decreased catalase activity while increasing superoxide dismutase activity. These findings indicate that IMD has the potential to significantly impact the development of bees and their colonies by disrupting vital organs responsible for normal physiological functioning and overall activities of individuals. Oxidative stress, which was detected at different stages of bee development, may induce lipid, protein, and DNA oxidation, leading to cell death.

**Graphical Abstract:**

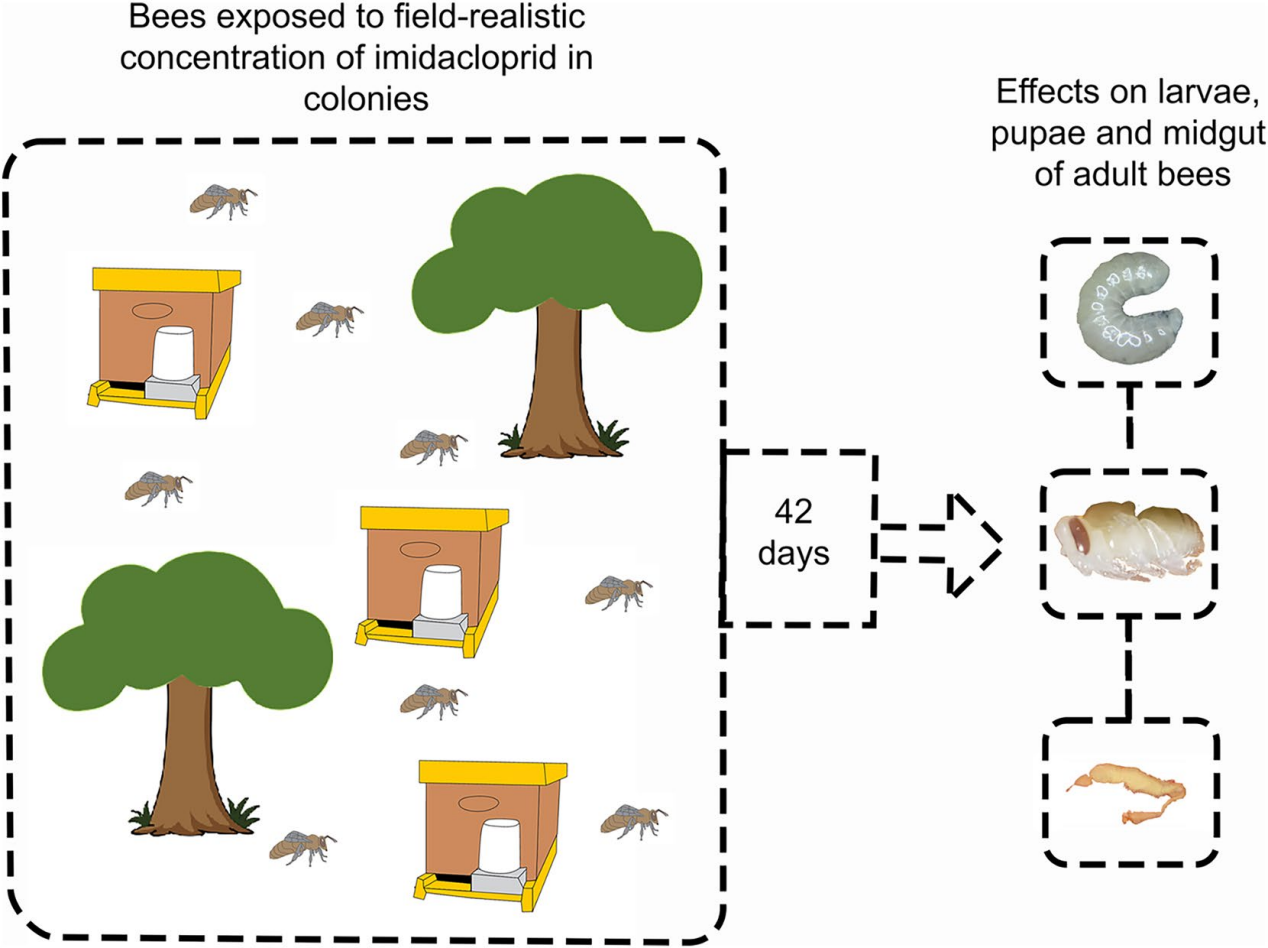

## Introduction

Pollination is an essential ecosystem service performed by several insects on different agricultural crops of economic interest worldwide [32, 59]. In Brazil, native bees and honey bees are responsible for pollinating 92% of crops, which contributes to approximately US$825 million in food production [70]. Among these pollinators, the *Apis mellifera* L. bee is the primary managed pollinator for food crops [9].

When visiting flowers to collect nectar or pollen, bees may come into contact with a variety of insecticides [67]. Even at sublethal doses, these compounds may produce toxic effects on these beneficial insects [75]. Given that pollination is extremely important for the maintenance of ecosystem biodiversity and food security, there is growing concern about the harmful effects of insecticides on different bee species.

The insecticide imidacloprid (IMD) is widely used in commercial formulations for pest control [51]. IMD is a nitro-substituted neonicotinoid compound that acts as an agonist of nicotinic acetylcholine receptors (nAChRs) in the brain and central nervous system of insects [66]. For bees, it causes changes in the normal functioning of the nervous system, resulting in symptoms such as hyperexcitation, tremors, and paralysis [37]. In severe cases, it can even lead to death [45, 67]. In the European Union, the use of neonicotinoids, including IMD, in outdoor crops has been banned since 2018 due to their harmful effects on bees and other pollinators [20]. In Brazil, the Brazilian Institute of the Environment and Renewable Natural Resources (IBAMA) and the Ministry of Agriculture, Livestock, and Supply (MAPA) establish guidelines for pesticide assessments, including pollinator risk. Whether a pesticide is considered a threat to pollinators and fails the analysis, it cannot be registered [6]. Both agencies work closely to ensure pesticide registration adheres to local regulations and international standards. Post-registration monitoring is conducted to ensure continued compliance with safety and environmental standards. However, in Brazil, the use of neonicotinoids, including IMD, is still permitted in agriculture for pest control. This discrepancy in regulations raises concerns about the potential threat to bee populations, as the parent compound of IMD is highly toxic to bees, and its metabolites are either equally or less toxic.

Bees can become contaminated with IMD by collecting and consuming pollen or nectar contaminated to this compound [42]. Pesticides are spread among colony members by trophallaxis [64], accumulating in larvae and adult bees causing behavioral, morphophysiological, and developmental changes in exposed individuals [15, 19, 50, 55]. Neonicotinoids such as IMD may also affect the immunocompetence of adult bees, making them more susceptible to diseases [11]. Furthermore, this class of insecticides may damage key organs such as the midgut and fat body, both of which are essential for the proper functioning of the whole organism [15].

In addition to the morphophysiological changes observed in bees, neonicotinoids can induce a state of oxidative stress in vertebrates and invertebrates [69]. Studies have shown that animals exposed to these insecticides have higher production of reactive oxygen (ROS) and nitrogen (RNS) species, as well as changes in enzymatic and non-enzymatic antioxidant defense mechanisms [4, 27, 34, 68, 69]. Imbalance between oxidants and antioxidants results in oxidative stress in living organisms. Oxidative stress may damage different molecules and cell structures, including lipids, proteins, and DNA, ultimately leading to cell death [27, 34], Balieira et al., 2015; Balieira et al., 2010; [4, 34, 39, 68, 69].

IMD residues in plant nectar and pollen pose a threat to bee larvae [42, 74]. The evaluation of *A. mellifera* bees with a realistic field concentration of IMD confirmed that the compound promoted histopathological damage in the larval midgut [14]. IMD caused a marked dose-dependent delay in larval development, characterized by reductions in body mass, width and growth index [48]. Next-generation sequencing indicated that sublethal IMD treatment during the larval stage caused changes in gene expression in larvae, pupae, and adults, indicating a prolonged sublethal effect on bee development [18].

Most studies assessing the effects of insecticides on bees use analytical standards or active ingredients [16, 19, 21, 54, 71]. However, the commercial formulation containing the inert ingredients, which is used by farmers in the field, has not been evaluated in most studies. Commercial products include, for instance, co-formulants that protect the active ingredient from degradation, increase its absorption by plants, and thereby, enhance its effectiveness [56]. In fungicide products, co-formulants were shown to increase the toxicity and effectiveness of the active ingredient [40].

When testing an acute oral dose of the fungicide product Amistar, it was identified that the alcohol ethoxylate co-formulant caused 30% mortality and promoted significant melanization of the midgut of *Bombus terrestris audax* bees [63]. The evaluation of the complete formulation of the fungicide Pristine and its active ingredients separately in *A. m. carnica* and *A. m. ligustica* bees revealed that the commercial product impaired the memory of the bees, while the active ingredients alone did not. This indicates that the inert ingredients are responsible for the effect [26].

Therefore, this study was conducted to test the hypothesis that imidacloprid, both in the form of active principle and in the form of the commercial product Evidence 700 WG^®^, could induce oxidative damage in larvae and pupae of Africanized bees (*A. mellifera*). Additionally, we aimed to investigate morphological and histological alterations in the fat body and midgut of larvae, as well as the midgut of adult bees. Through this study, we sought to answer the following questions: (1) Is there a difference between the effects of imidacloprid in the form of active principle and the commercial product Evidence 700 WG^®^? (2) How does imidacloprid affect the redox metabolism of whole larvae and pupae, and also the structure of larvae and the midgut of adult bees?

## Material and methods

### Experimental design

The apiaries were installed at the Iguatemi Experimental Farm, State University of Maringá (23°25′S 51°57′W, 550 m a.s.l.), Paraná, Brazil. A total of 18 *A. mellifera* colonies housed in standard Langstroth hives were standardized prior to the start of the experiment. The colonies were standardized to include: 1 frames with food (honey and bee bread), 1 frames with drawn comb, 1 frames with open brood, and 2 frames with capped brood.

Colonies showed no symptoms of disease or parasite infection. Sister queens from the same lineage were used so as to ensure homogeneity of genetic background among treatment groups.

The experiment was carried out from October to November 2019 for 42 days, encompassing two complete development cycles. The 18 colonies were distributed into three treatments according to a completely randomized design with six replicates per treatment, as follows: (i) an untreated control group, (ii) a treatment group exposed to the commercial formulation Evidence^®^ 700WG (IMD_CF_) (Brazilian Ministry of Agriculture, Livestock, and Food Supply registration No. 06294, 700 g a.i. kg^−1^), and (iii) a treatment group exposed to IMD active ingredient (IMD_AI_) (98.2% purity; Sigma–Aldrich, UK; CAS 138261-41-3).

Colonies were monitored every 15 days. Management was carried out in all colonies to visually check the behavior and activity of the bees, such as pollen and honey storage, queen posture and the need to include a supernest containing more frames with wax.

### Insecticide dilution and application procedures

Evidence^®^ 700WG and IMD active ingredient were separately dissolved in distilled water and stored in amber bottles at 4 °C throughout the experimental period. The IMD concentration used in the study was 1 µg L^−1^, selected on the basis of field-realistic concentrations found in pollen and nectar in previous studies (0.7–10 µg L^−1^) [23, 25]. Insecticide treatments were delivered through oral exposure using separate stock solutions of spiked syrup for each replicate. IMD_AI_ and IMD_CF_ were mixed with syrup (2:1 water/sugar) to a final concentration of 1 µg L^−1^ IMD. The control group received syrup only. About 300 mL of syrup was provided to each colony every 3 days, between 12:00 and 14:00 h, in a Boardman feeder installed outside the hive.

### Collection of bee individuals at different developmental stages

For the collection of larval specimens, a central honeycomb with empty alveoli for egg laying was demarcated in the nest. After a 6–8-day egg-laying period, larvae aged 3–5 days were sampled. Brown-eyed pupae were selected for analysis. Adult bees were randomly collected from within the colony.

### Morphological analysis

#### Light microscopy

After 42 days of experiment, 3–5-day-old larvae (*n* = 90 by treatment) and adult bees (*n* = 90 by treatment) were collected, cold-immobilized for 2 min, and dissected to extract the midgut. Specimens were fixed in Bouin solution for 12 h, dehydrated in an increasing ethanol series (70%, 80%, 90%, and 100%), cleared with xylol, immersed in histological paraffin (Alpha 580), and cut into 6 µm sections using a microtome (Leica RM 2250). Histological sections were stained with hematoxylin and eosin [43]. Images were captured using a Leica light microscope coupled to an Olympus PMC 35 B digital camera.

#### Scanning *electron* microscopy (SEM)

Larvae aged 3–5 days (*n* = 90 by treatment) and adult individuals (*n* = 90 by treatment) were collected directly from the hive frame. The midgut was dissected and fixed in Bouin solution for 12 h. After this period, samples were dehydrated in an increasing ethanol series (70%, 80%, 90%, and 100%) and subjected to critical point drying (BAL-TEC CPD-030). Dried samples were mounted on stubs, sputter-coated with gold (BAL-TEC SCD-050), and examined under a scanning electron microscope (FEI Quanta 250).

### Analysis of oxidative stress in larvae and pupae

At the end of the 42-day experimental period, 36 larvae (3–5-day-old) and 36 brown-eyed pupae were collected from each treatment, placed in cryotubes, frozen in liquid nitrogen, and stored in a freezer at − 80 °C until analysis. Superoxide dismutase (SOD) activity, catalase (CAT) activity, total antioxidant capacity, nitrite content, and carbonylated protein content were determined on whole samples [1, 10, 12, 44, 47, 52].

Reduced glutathione (GSH), a potent non-enzymatic antioxidant, was determined according to the method described by Ellman [28], with some modifications. About 100 mg of whole larvae and pupae were added separately into test tubes containing 1000 µL of 50 mM Tris buffer (pH 7.5). Samples were homogenized using a Dounce homogenizer until complete dissociation was achieved. Then, homogenates were centrifuged at 3000 rpm and 4 °C for 10 min. Supernatants were collected into clean microtubes and used as crude extracts.

For GSH determination, 225 µL of 1 M potassium phosphate buffer (pH 7.4), 60 µL of crude extract, and 15 µL of 5,5′-dithiobis-(2-nitrobenzoic acid) (DTNB, Sigma–Aldrich) at 10 mM were pipetted, in duplicate, into the wells of a 96-well microplate. Absorbance readings were taken at 412 nm using a microplate reader (VersaMax^™^, Molecular Devices). A standard curve of l-glutathione (1 mM, Sigma–Aldrich) was constructed to verify whether sample absorbances were within the linear portion of the curve. A standard curve of l-cysteine (0.5 mM, Sigma–Aldrich) was used to calculate the cysteine factor for estimation of GSH contents. GSH content was calculated as follows: GSH = (Sample absorbance × Cysteine Factor)/Sample weight. Results are expressed as µmol GSH g^−1^/protein.

### Data analysis

Morphological data were analyzed qualitatively. Oxidative stress data from larvae and pupae were tested for normality using the Shapiro–Wilk test and subjected to analysis of variance (ANOVA) followed by Tukey's test (*p* < 0.05) for comparison of means [60].

## Results

### Morphological changes in larvae

In the experiment, the colonies exposed to IMD were visually monitored. During continuous management and feeding, the bees exhibited normal behavior and activity.

**Fig. 1:**
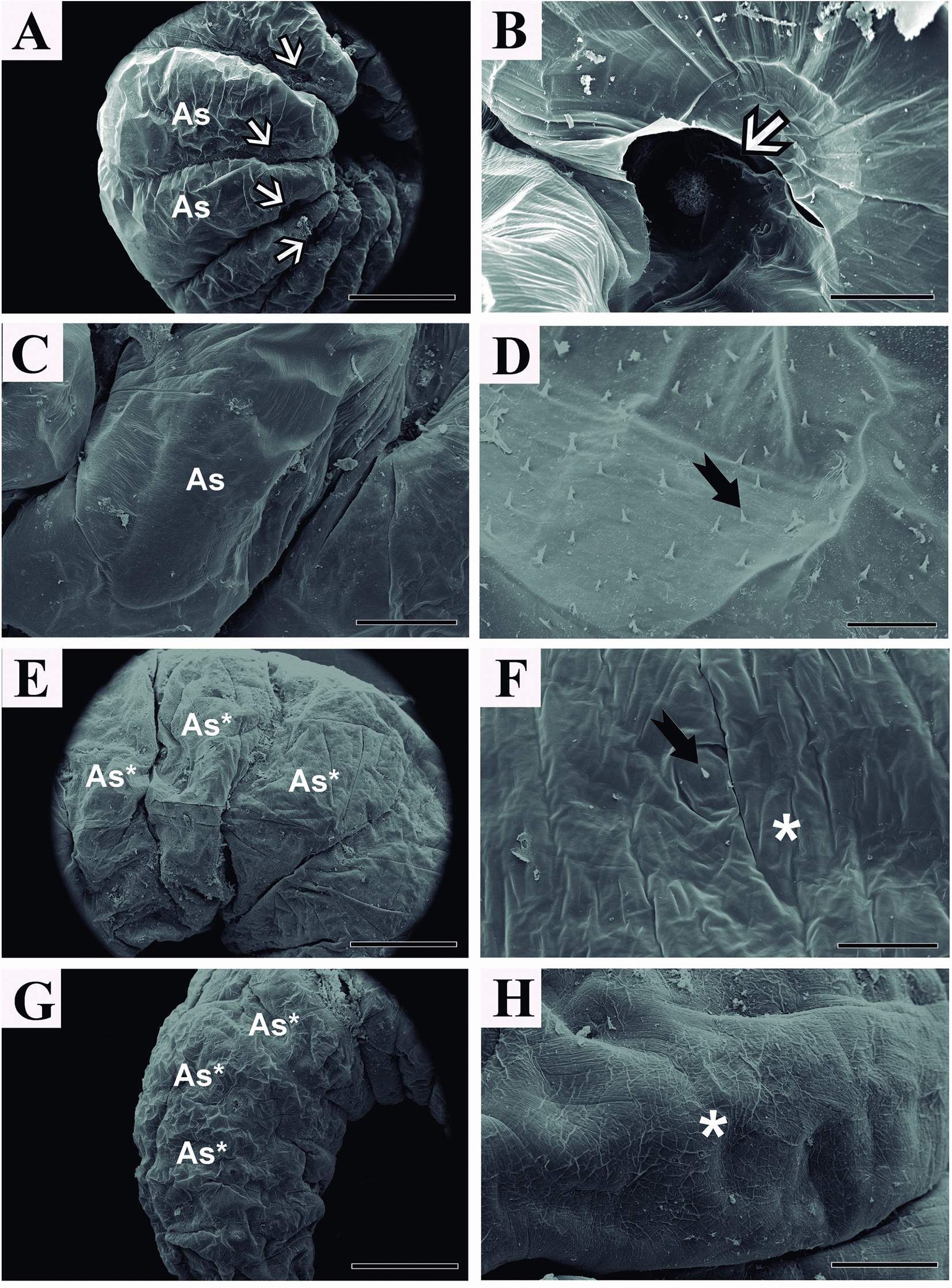
Scanning electron microscopy showing the overview of *A*. *mellifera* larvae after oral exposure to inidacloprid. **A**, **B**, **C**, **D** = control. **E** and **F** = commercial formulation IMD; **G** and **H** = active ingredient IMD. As = abdominal segment; Sa* = deformed abdominal segment; * = integument rugose; ⇒ = spiracles; →  = spicules. Scale; **A**, **E**, **G** = 1 mm; **B** = 100 µm; **C**, **H** = 200 µm; **D**, **F** = 30 µm

In the larvae, we attempted to analyze both the external (SEM) and internal parts of the insects (midgut and fat body—Light microscopy). The larvae in the control group of *A. mellifera* bees displayed a white and vermiform appearance, lacking legs, wings, antennae, or external. Externally, the body was covered by integument composed of an undifferentiated cuticle, spicules projecting toward the surface, and cuticular constrictions dividing the surface into segments or sclerites united by intersegmental membranes. The larval body is composed of three thoracic segments and ten abdominal segments, presenting a ventrolateral suture and depressions where the spiracles are located (Figs. 1A–D, 3A and B).

**Fig. 2:**
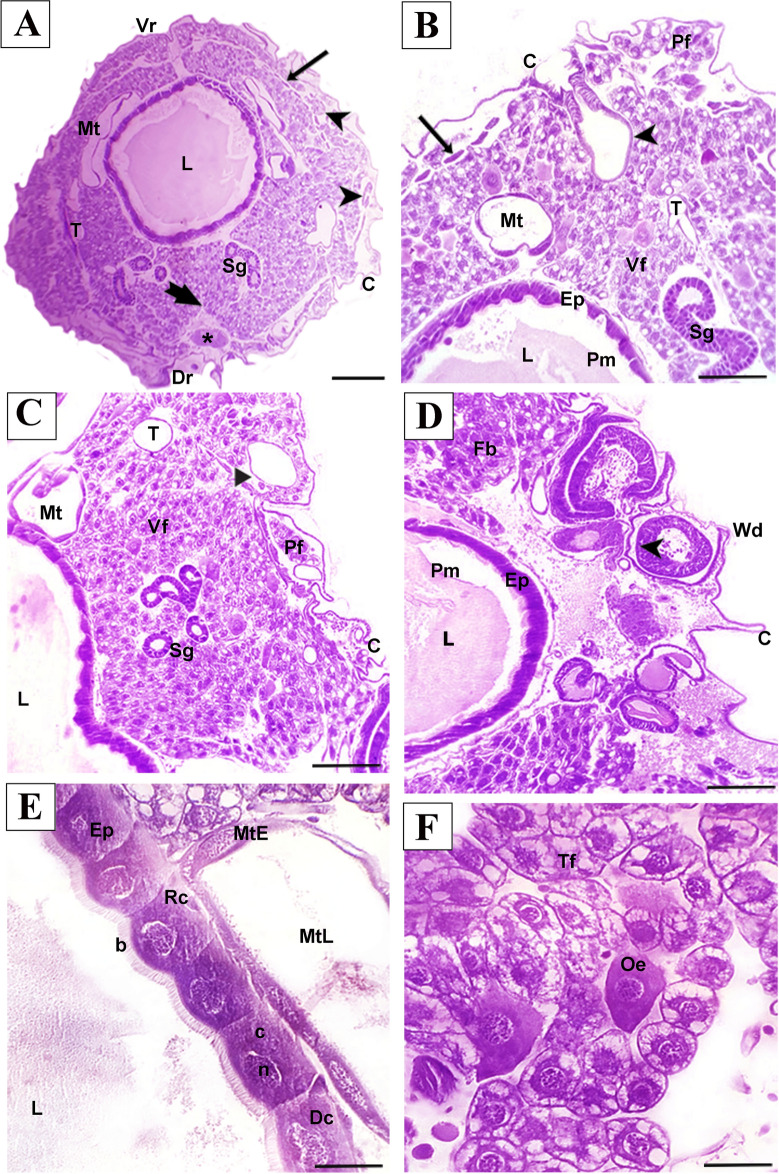
Photomicrograph of cross-section of *A*. *mellifera* larvae not exposed to imidacloprid. Md = midgut; Dr = dorsal region; Vr = ventral region; Sg = salivary gland; T = tracheola; Mt = Malpighian tubule; C = cuticle; * = abdominal ganglia; L = lumen; Pf = parietal fat body; Vf = visceral fat body; Pm = peritrophic membrane; Ep = epithetlium; Sg = salivar glands; Fb = fat body; Wd = imaginal wing discs; b = striated border; Rc = regenerative cells; N = nucleus; C = cytoplasm; Dc = digestive cell; MtE = Malpighian tubule epithelium; MtL = Malpighian tubule lumen; Oe = oenocytes; Tf = trophocytes; ⇒ = vental sinus; ⇒spiracle; →  = central nerve cord; ⊳ = intersegmental musculature. Staining: Hematrxylin-eosin. Scale: **A** = 100 µm; **B**-F = 20 µm

**Fig. 3:**
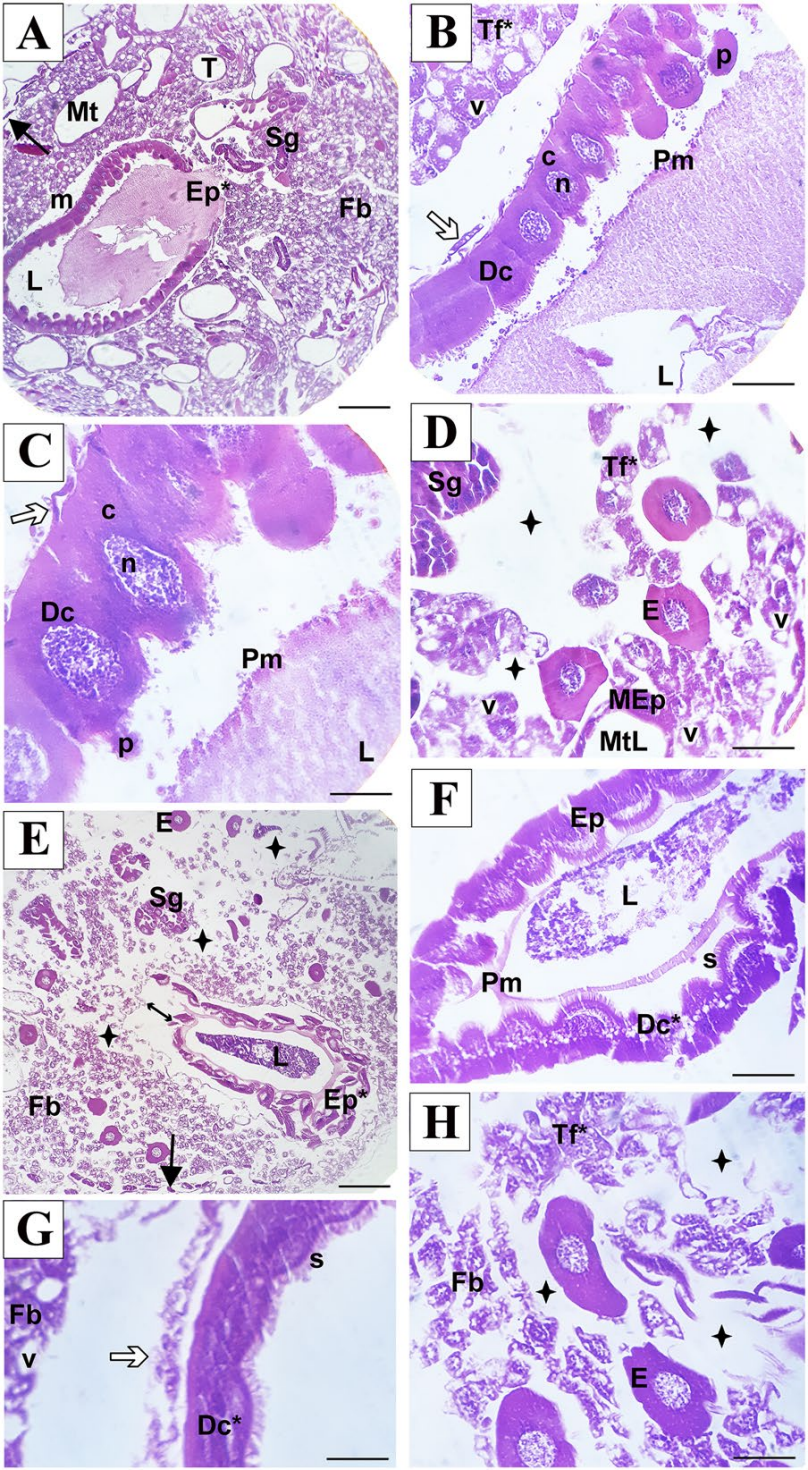
Photomicrograph of cross-section of A. *mellifera* larvae after oral exposure to imidacloprid. **A**-**D** = commercial formulation IMD. **E**–**H** = active ingredient IMD. **A**, **E** = larva overview; **B**,**C**,**F**,**G** = larval midgut; D,H = fat body larval. *Ep = deformed epithelium; Sg = salivary gland; T = tracheola; Mt = Malpighian tubue; Fb = fat body; m = musculature; L = lumen; Tf* = reduced trophocyte; Pm = peritrophic membrane; Dc = digestive cell; v = vacuolized; c = cytoplasm; n = nucles; p = cytoplasmic protrusions; MEp = Malpighian tubule epithelium; MtL = Malpighian tubule lumen; E = oenocytes; Ep = epithelium; b = striated border: Dc* = deformed digestive cells; →  = central nerve cord; ⇒ = musculature loose; ${ + }$ = intercellular spaces. Staining: Hematoxylin–eosin. Scale: **A**, **E** = 100 µm; **B**, **C**, **D**, **F**, **G**, **H** = 20 µm

Spiracles appeared as outward-projecting circular apertures (Figs. 1B, 3B). Histologically, the larvae had adipose tissues filling the body cavity and immature midgut in the central region, in addition to tracheoles, Malpighian tubules, abdominal ganglia, ventral sinus, abdominal salivary gland, and central nerve cord (Fig. 2A). It was also possible to observe imaginal discs formed by embryonic cells organized into distinct groups (Fig. 2D).

The midgut of control bees larvae was composed of a simple epithelium resting on a thin layer of muscles and basal lamina (Fig. 2A–E). The short and cuboid digestive cells of the midgut had a spherical nucleus in the apical cytoplasm and were surmounted by a striated border. Small regenerative cells were observed in the basal region of the epithelium (Fig. 2E). A peritrophic membrane separated the lumen into endo and ectoperitrophic spaces (Fig. 2B and D). Malpighi tubules were thin and long, extending toward the anterior region until reaching the thoracic region. The simple epithelium was composed of cells with acidophilic cytoplasm and basophilic nucleus. The lumen was well defined (Fig. 2C and E).

The fat body was distributed throughout the larva's body, filling two compartments divided by intersegmental musculature, namely the parietal compartment (smaller cells), which is located below the integument between interstitial muscles, and the perivisceral compartment (larger cells), surrounding organs (Fig. 2B and C). Two types of cells were identified, trophocytes and oenocytes. Trophocytes were abundant, spherical cells surrounded by basal lamina in direct contact with the hemolymph. Oenocytes were located between trophocytes and were identified as large, spherical, irregular cells with granule-free acidophilic cytoplasm (Fig. 2F). The absence of visible abnormalities in untreated larvae validates their use as a control.

Bee larvae exposed to IMD_CF_ and IMD_AI_ lacked external cuticular constrictions and showed undefined abdominal segments, closed spiracles (Fig. 1E and G), and reduced number or absence of spicules. In IMD_AI_-treated larvae, the integument was rugose (Fig. 1F and H).

Loosened musculature lining the midgut was observed in all bees analyzed in the IMD_CF_-treated larvae group. Digestive cells showed enlarged intercellular spaces and granular cytoplasm with cytoplasmic protrusions extending into the lumen. The regenerative cells were not observed (Fig. 3A–C).

In all evaluated bees, larvae treated with IMD_AI_ exhibited detachment of the midgut epithelium in relation to the loose musculature (Fig. 3E and F), formation of intercellular spaces associated with epithelial disorganization (Fig. 3E, G, and H), digestive cells with granular and vacuolized cytoplasm detached into the lumen (Fig. 3F), and absence of regenerative cells (Fig. 3F and G). In these individuals, the fat body was more vacuolized and had a lower amount of trophocytes, as well as increased intercellular spaces and cell volume (Fig. 3D and H). Oenocytes were unaltered in larvae from both treatments.

### Morphological changes in the midgut of adult workers

**Fig. 4:**
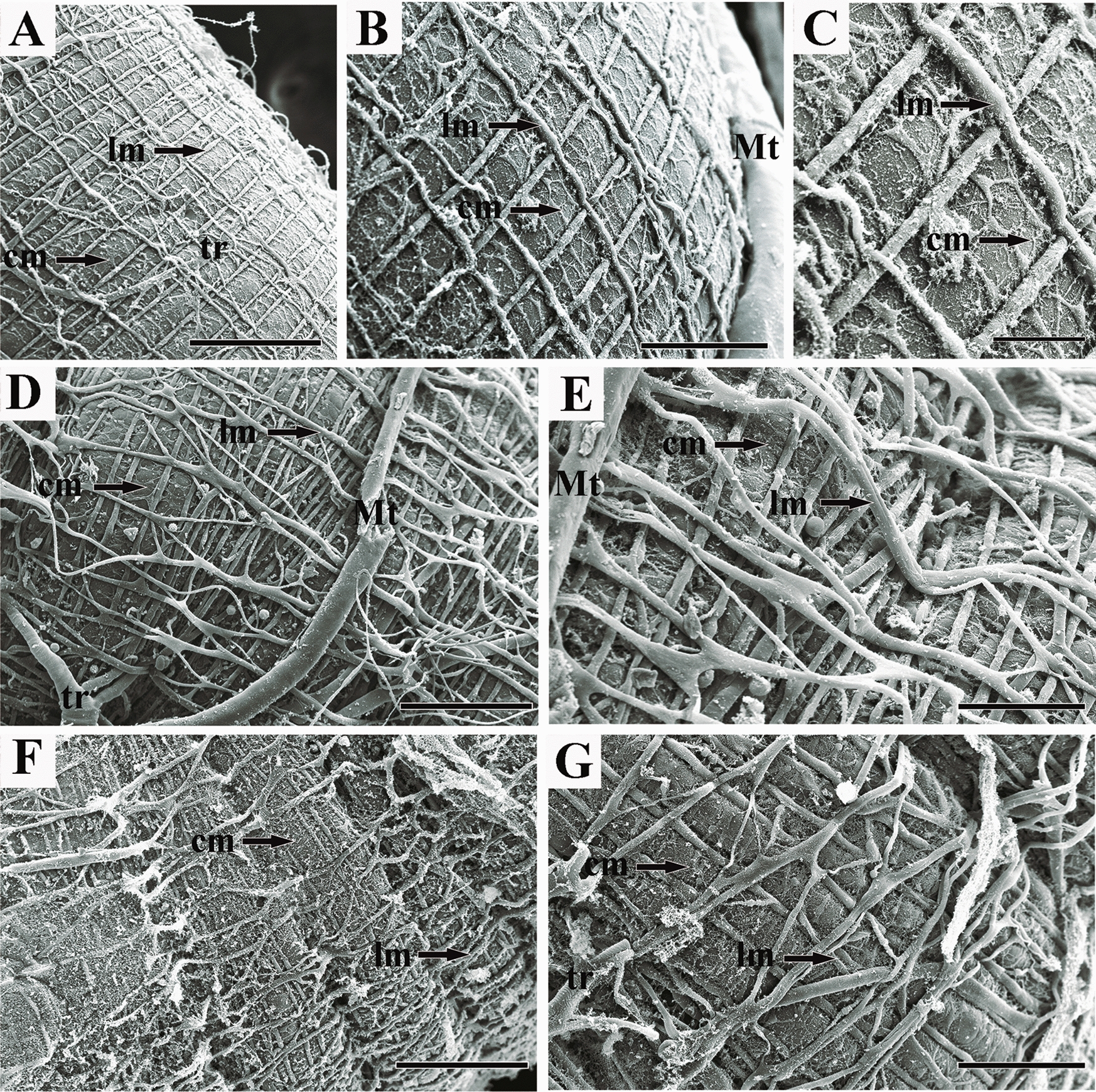
Scaning electron microscopy showing the midgut of *A*. *mellifera* workers after oral exposure to imidaclaprid. **A**, **B** and **C** = control; **D** and **E** = commercial formulation IMD; **F** and **G** = active ingredient IMD. Im = longitudinal musculature; cm = circular musculature; tr = tracheolas; Mt = Malpighian tubule. Scale: **A**, **D**, **F** = 200 µm; **B**, **E**, **G** = 100 µm; **C** = 50 µm

**Fig. 5:**
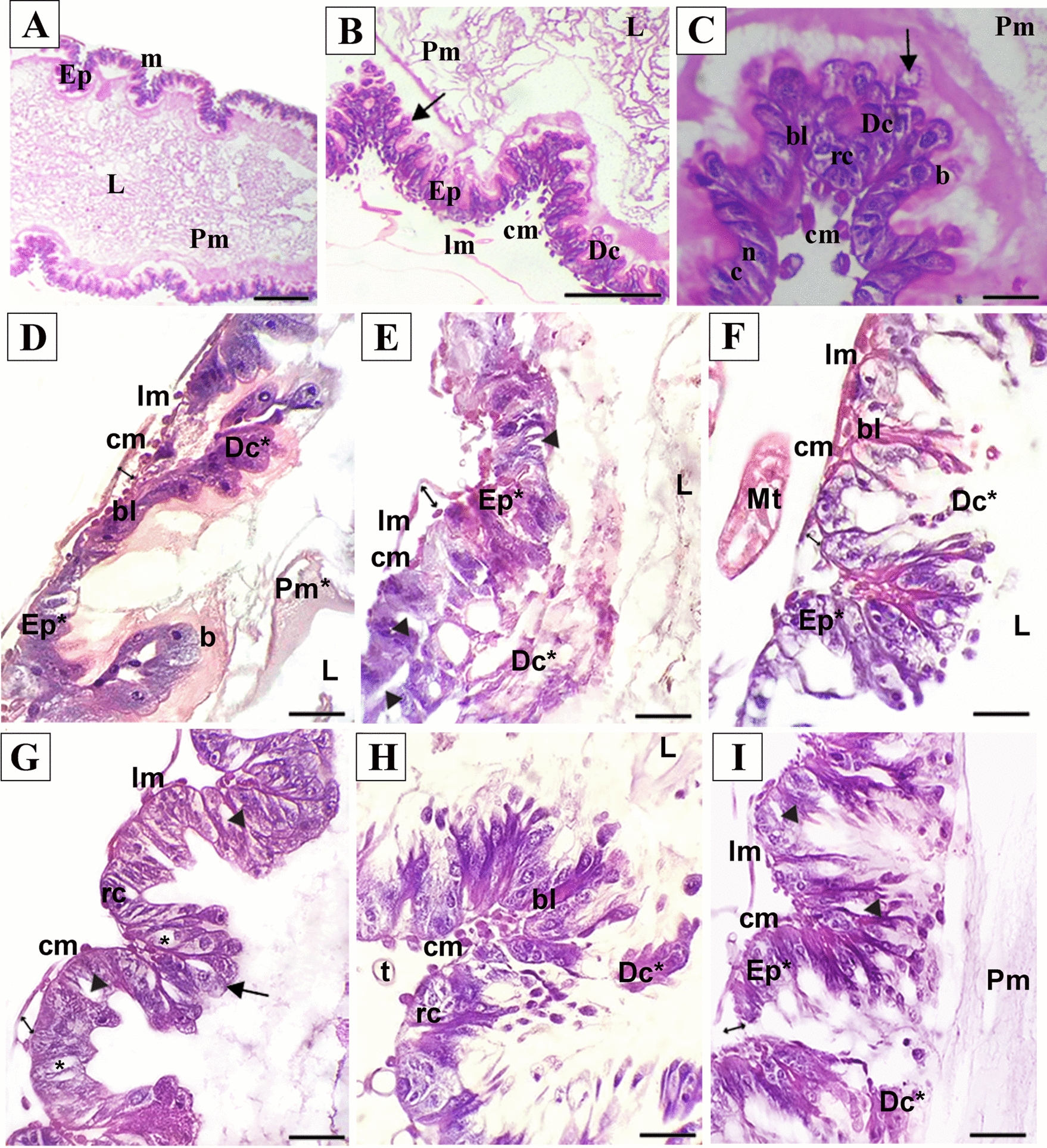
Photomicrograph of the longitudinal section of the midgut of *A*. *mellifera* workers after oral exposure to imidacloprid. **A**, **B** and **C** = control; **D**, **E** and **F** = commercial formulation IMD; **G**, **H** and **I** = active ingredient IMD. Ep = epithelium of midgut; m = musculature; L = lumen; Pm = peritrophic membrane; cm = circular musculature; Im = longitudinal musculature; Dc = digestive cell; bl = basal lamina; rc = regenerated cells; n = nucleus; c = cytoplasm; b = striated border; Ep* = deformed epithelium; Dc* = release of digestive cells to lumen; Pm* = degenerated peritrophic membrane; Mt = Malpighian tubule; * = vacuolated digestive cells; t = traqueiolas; →  = cytoplasm protrusion; ↔  = loosening of the longitudinal musculature and separation from the circular musculature; ⊳ = intercellular spaces. Staning: Hematoxylin–eosin. Scale: **A** = 100 µm; **B**-**l** = 20 µm

In adult insects, we evaluated the musculature externally (SEM) and the midgut internally (Light microscopy). The midgut of control adult bees was composed of a cylindrical, thick, long tube, with the outer surface covered by two muscular layers: circular fibers arranged internally forming circular folds and external longitudinal fibers (Fig. 4A–C). The midgut consisted of a simple epithelium with digestive and regenerative cells resting on the basal lamina (Fig. 5A–C). Digestive cells were cylindrical, exhibiting basophilic cytoplasm, with the nucleus located in the basal to median region (Fig. 5B). We observed cytoplasmic protrusions in the apical region, indicating the occurrence of secretion, and long striated edges that extend toward the lumen covered by a thick layer of amorphous material (Fig. 5B–C).

Regenerative cells from the midgut of adult bees had a spherical shape and bulky nucleus, occupying most of the cytoplasmic volume. They were grouped into nests located near the basal lamina (Fig. 5B–C). In the lumen, the peritrophic membrane consisted of numerous overlapping lamellar layers delimiting endo- and ectoperitrophic spaces, separating the epithelium from luminal contents (Fig. 5A–B). These characteristics confirm the preservation of normal midgut morphology in control adult bees.

Exposure of adult workers to IMD_CF_ and IMD_AI_ resulted in epithelial disorganization in the midgut (Fig. 5D–I), musculature loosening, and separation of the circular musculature from the longitudinal musculature (Figs. 4D–E and G, 5D–G). In bees exposed to IMD_CF_ and IMD_AI_, there was detachment of the basal lamina from the musculature. Furthermore, vacuolized digestive cells with reduced striated edges were found to be released in the lumen (Fig. 5D–F). Apical secretions were observed only in IMD_AI_-treated bees, indicating secretory activity of these cells after exposure (Fig. 5G). Regeneration nests were not observed in the midgut of bees treated with IMD_CF_ (Fig. 5D–F); however, they were found in the basal epithelial in bees exposed to IMD_AI_ (Fig. 5G–H). Structural remnants of the peritrophic membrane were observed in subjects exposed to IMD_CF_ (Fig. 5D–E), whereas IMD_AI_ treatment resulted in a more organized peritrophic membrane in the midgut lumen (Fig. 5H).

### Analysis of oxidative stress in larvae and pupae

**Fig. 6:**
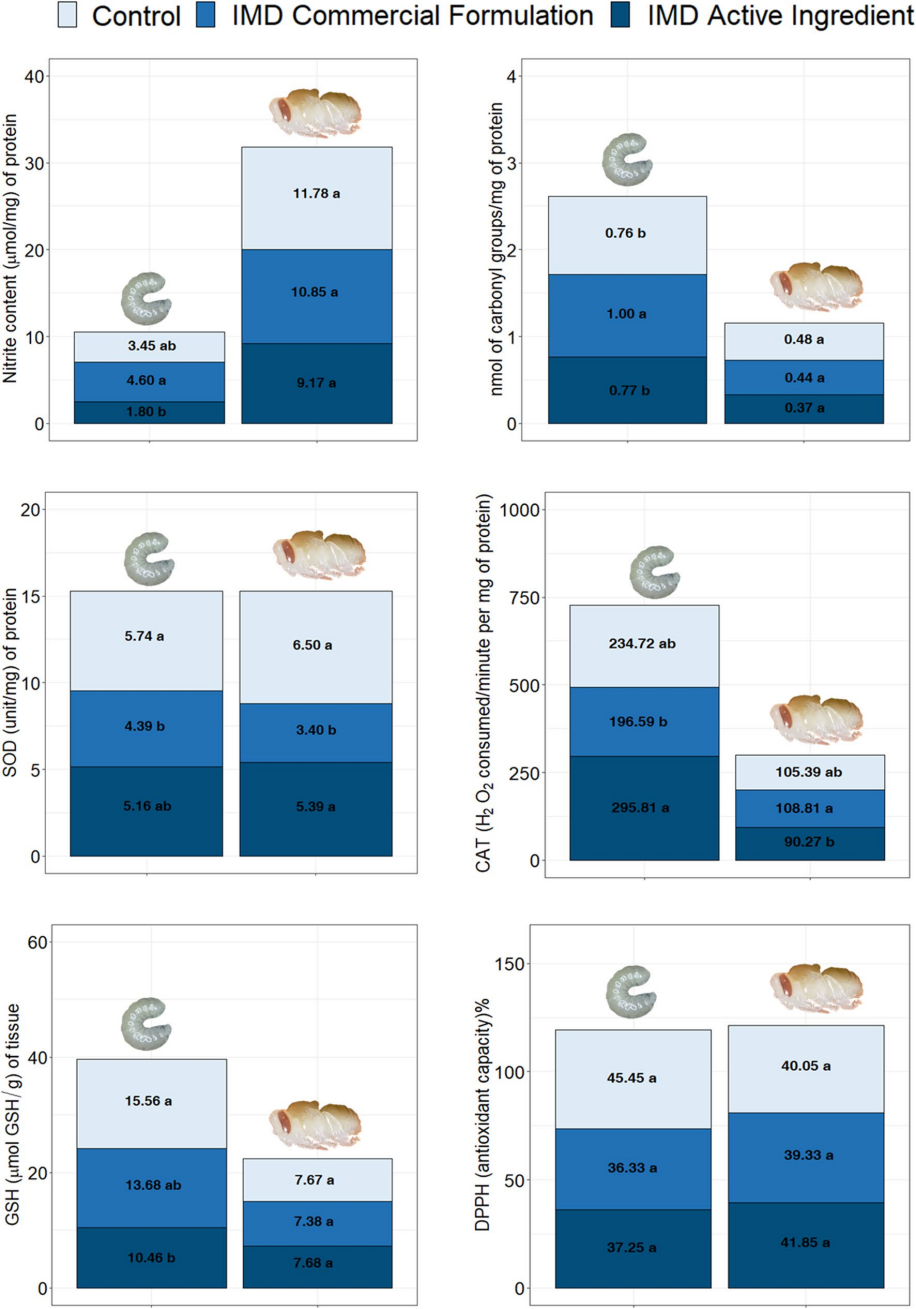
Effects of exposure to the commercial formulation IMD and active ingredient IMD on the redox status of larvae and pupae (*Apis mellifera*). **a**, **b** Means in the same column with different letters are significantly different by Tukey’s test (p-value < 0.05)

The homoscedasticity of variances was confirmed using the Bartlett test (Table 1). The results of the F statistics and degrees of freedom of the Anova test are described in Table 2. In larvae, both treatments influenced nitrite and carbonylated protein contents, SOD and CAT activities, and GSH content. Larvae exposed to IMD_CF_ had higher nitrite and carbonylated protein contents and lower SOD and CAT activities. IMD_AI_ treatment led to an increase in SOD and CAT activities in larvae. Larvae exposed to IMD_CF_ and IMD_AI_ had the lowest GSH contents. In general, there were no significant effects of treatments on total antioxidant capacity (*p* > 0.05). The results for nitrite and carbonylated protein contents, SOD and CAT activities, GSH content, and total antioxidant capacity in larvae and pupae exposed to IMD are depicted in Fig. 6.

**Table 1:** Bartlett test results for homecedasticity of variances

Variables	Bartlett test* P-values ($X^{2}$: Bartlett’s test statistic, df: degrees of freedom)	
	Pupae	Larvae
Nitrite content	0.56 ${(\chi }^{2}=1.14, \text{df}=2)$	0.85 ${(\chi }^{2}=0.31, \text{df}=2)$
SOD	0.61 ${(\chi }^{2}=0.98, \text{df}=2)$	0.64 ${(\chi }^{2}=0.87, \text{df}=2)$
GSH	0.73 ${(\chi }^{2}=0.62, \text{df}=2)$	0.38 ${(\chi }^{2}=1.89, \text{df}=2)$
nmol of Carbonyl	0.98 ${(\chi }^{2}=0.03, \text{df}=2)$	0.55 ${(\chi }^{2}=1.17, \text{df}=2)$
CAT	0.80 ${(\chi }^{2}=0.43, \text{df}=2)$	0.05 ${(\chi }^{2}=5.90, \text{df}=2)$
DPPH	0.63 ${(\chi }^{2}=0.91, \text{df}=2)$	0.44 ${(\chi }^{2}=1.63, \text{df}=2)$

^*^H_0_: All k population variances are equal, i.e., the null hypothesis is rejected if (p- value < 0.05)

**Table 2:** F statistics and degrees of freedom of the Anova test

Variables	Anova test F’s test statistic (dt: degrees of freedom)	
	Pupae	Larvae
Nitrite content	2.88($\text{df}=2)$	19.68($\text{df}=2)$
SOD	19.97($\text{df}=2)$	21.19($\text{df}=2)$
GSH	0.19($\text{df}=2)$	20.77($\text{df}=2)$
nmol of Carbonyl	0.50 $(\text{df}=2)$	20.49 $(\text{df}=2)$
CAT	$20.49(\text{df}=2)$	19.74 $(\text{df}=2)$
DPPH	0.17 $(\text{df}=2)$	0.82 $(\text{df}=2)$

In pupae, treatments significantly influenced SOD and CAT activities. IMD_AI_-treated pupae had higher SOD and lower CAT activities. Treatments did not influence nitrite content, carbonylated protein content, GSH content, or total antioxidant capacity (*p* > 0.05).

## Discussion

The concentration of IMD used to assess toxicity in *A. mellifera* (1 µg L^−1^) was lower compared to previous studies conducted by Wu-Smart and Spivak [72] on the same species in field colonies, using concentrations of 10, 20, 50, and 100 µg of IMD active ingredient. In our research, all colonies, including those exposed and unexposed to IMD, had unrestricted access to the field. Thus, considering that control colonies did not exhibit any changes in the assessed parameter, the observed alterations can be attributed to the consumption of the neonicotinoid-contaminated diets.

In bee larvae exposed to IMD, the presence of rugose integument, changes in the conformation of larval segments, and obstruction of spiracles may be indicative of developmental changes, which may result in the death of bees while still in the larval stage. Associated with these alterations, the absence and/or reduction in the number of spicules was observed in both treatments. Additionally, the absence and/or reduction in the quantity of spicules observed in both treatments may indicate a failure in the protection mechanism against IMD exposure [17, 36], since the control shows a large number of these structures.

The larval fat body was also affected by both IMD treatments. The reduction and vacuolation of trophocytes might have negatively impacted compound metabolization, harming the insect [2]. Given that the fat body is an important endocrine organ and is used for energy storage, prolonged exposure to the insecticide could harm insects, resulting in insufficient energy for molting and completing development [24].

From this perspective, exposure to neonicotinoid compounds can disrupt both the internal functioning and population dynamics of a colony. Low concentrations of IMD are likely to compromise survival conditions. Notably, since bee larvae can consume up to 160 µL of larval food before pupation [3], it is plausible that *A. mellifera* larvae in this study were affected by IMD, as indicated by the significant morphological changes observed.

In adults, exposure to IMD promoted changes in midgut musculature and epithelial disorganization. The presence of vacuolized digestive cells was observed in all IMD treatments, as also reported in the midgut of *A. mellifera* exposed in vitro to different doses of the active ingredient of IMD [15] and that of thiamethoxam [57], as well as in other insects exposed to thiamethoxam and IMD, Ameen et al., 2020). Similarly, reduction or loss of striated edges in the apical portions of digestive cells was observed in *A. mellifera* exposed to 5 and 20 ppb IMD under laboratory conditions [37].

Epithelial disorganization, as well as alterations in digestive cells, is indicative of cellular degeneration processes induced by IMD. In light of the important role of digestive cells, such as in enzyme secretion and nutrient absorption [61], their loss can lead to nutritional deficiency, compromising important physiological processes, affecting bee survival, and possibly resulting in colony decline.

Digestive cell degeneration was more severe in bees exposed IMD_CF_. This is because the absence of regenerative cells in the midgut made it difficult to replace damaged cells. In IMD_CF_-treated individuals, cell damage and death occurred at a faster rate, and regenerative cells were depleted within 42 days of exposure. The long-term effects of these changes are not known. If regenerative cells are depleted, the intestinal epithelium may have difficulty recovering, since these cells are essential for the renewal and repair of damaged tissue. However, the ability to recover will depend on the severity of the damage and the presence of alternative regeneration mechanisms in the insect's body [31, 65].

The lower involvement of the midgut of bees exposed to IMD_AI_ compared with IMD_CF_ can be attributed to the presence of a more organized peritrophic membrane in the former, acting as a barrier to minimize the amount of contact between the intestinal epithelium and insecticide compounds. As a result, regenerative cells could maintain their ability to divide and differentiate into new digestive cells to reorganize the epithelium.

The redox system of larvae and pupae exposed to 1 µg L^−1^ IMD was impaired. Many of the changes observed in the larvae might be related to oxidative stress caused by the neonicotinoid. In general, neonicotinoid insecticides, when metabolized, can cause oxidative stress by generating ROS, such as superoxide (O_2_^•−^) and hydrogen peroxide (H_2_O_2_), and RNS, including nitric oxide and peroxynitrite, in quantities greater than what the cellular antioxidant defense system is capable of eliminating [27, 34, 69].

In this study, treatment with IMD_CF_ and IMD_AI_ promoted oxidative stress in *A. mellifera* larvae and pupae. Larvae exposed to IMD_CF_ had higher nitrite and carbonylated protein (measure of protein oxidation) contents. Nitrite is one of the two primary, stable, and non-volatile products of nitric oxide degradation [7, 38]. Here, it was used to assess the formation of nitric oxide (RNS) in bees as a potential cause of oxidative damage of important biomolecules. Protein carbonylation is an oxidative modification induced by ROS and RNS (including nitrite) capable of altering biological functions [8, 35]. This protein modification stems from the oxidation of some amino acid residues, initiated by ROS and RNS, which directly attack the protein, producing highly reactive carbonyl derivatives by oxidizing the side chains of amino acid residues [8, 35, 62]. Furthermore, RNS can oxidize proteins and alter their biological functions in other ways, such as nitration of amino acids [29]. Nitration can also be promoted by heme peroxidases and nitrite [8, 35].

IMD_CF_-treated larvae had lower SOD and CAT activities. To protect against oxidative damage, vertebrate and invertebrate organisms rely on at least two very efficient antioxidant defense mechanisms [22]. The first line of defense involves antioxidant enzymes capable of converting ROS/RNS into less reactive species with reduced cytotoxicity [41, 46]. Our findings indicate that the oxidative substances produced by exposure to IMD triggered an antioxidant response [33]. IMD_CF_ might have induced morphological changes and altered antioxidant production.

The long exposure of larvae to the neonicotinoid aggravated their state of oxidative stress, overloading the function of antioxidant enzymes, causing loss of function and/or inefficiency compared with the control group. The confirmed changes in the external and internal larval structures promoted by IMD may be related to the modifications found in nitrite and carbonylated protein levels, SOD and CAT activities, and GSH content.

Organisms were unable to counterbalance the production of oxidizing compounds (free radicals), which can damage various cellular constituents, leading to tissue and organ dysfunction [33]. Moreover, ROS might have inactivated SOD via oxidation [73]. The low CAT activity might have been due to inhibition by nitric oxide [5, 13] and other ROS, including superoxide anion and hydroxyl radical [58].

A second antioxidant defense mechanism used by animals is that mediated by non-enzymatic antioxidants, compounds capable of rapidly inactivating oxidizing substances that are harmful to the body, thereby preserving important biomolecules [53]. GSH is an example of a non-enzymatic antioxidant that reacts directly to eliminate reactive oxygen species (ROS) and reactive nitrogen species (RNS), thereby preventing or delaying the occurrence of various oxidative processes [46]. GSH is considered the most abundant endogenous non-protein thiol in the body, being essential for several functions, including redox signaling of cells and xenobiotic detoxification [49]. Here, it was found that IMD_CF_- and IMD_AI_-exposed larvae had low GSH contents, suggesting that GSH was being widely used in antioxidant defense mechanisms or that its synthesis was inhibited by IMD.

The low GSH content in exposed larvae might have contributed to the occurrence of oxidative damage. Thus, we suggest that larvae treated with IMD, particularly IMD_CF_, were experiencing oxidative stress. The reduction in SOD and CAT activities, combined with low GSH content, likely promoted an irreversible autocatalytic process, in which the production of oxidizing compounds increases, ultimately leading to cell death [58]. It is noteworthy that changes at the cellular level resulting from oxidative processes might have contributed to the occurrence of morphological damage in larvae.

In IMD_AI_-exposed pupae, there was an increase in SOD and a reduction in CAT activities. These findings suggest the onset of oxidative stress and an attempt to fight ROS. The increased SOD activity seems to have been sufficient to decrease the excess levels of superoxide produced during detoxification. However, the low CAT activity suggests inhibition of the enzyme by RNS [5, 13], and, therefore, the biological detoxification process initiated by SOD was likely not completed. Generation of toxic substances resulting from exposure to IMD_AI_ possibly exceeded the defense capacity of CAT.

Moreover, the alterations caused by IMD_CF_ may have been exacerbated by the inert ingredients present in the product. As expected for their effectiveness against pests, these inert compounds can enhance the toxicity of the active ingredient in the commercial formulation. However, studies have also revealed their detrimental effects on non-target organisms, including bees. For instance, they can impair crucial abilities such as olfactory learning, vital for foraging [56], and disrupt pupal emergence and melanization in *A. mellifera ligustica* [30]. Therefore, inert compounds should be incorporated in toxicity tests, as they may either amplify the effects of the active ingredient in commercial formulations or directly influence bee morphophysiology. This emphasizes the significance of evaluating the toxicity of commercial products used in the field, given the heightened risk associated with the presence of inert compounds that can compromise colony survival.

## Conclusions

The internal and external morphology of *A. mellifera* was affected by oral exposure to both an IMD commercial formulation and its active ingredient. The effects of IMD_CF_ were found to be more severe. Assessment of the larval fat body and oxidative state indicated alterations that may have long-term irreversibility, potentially leading to colony mortality. *A. mellifera* pupae experienced oxidative stress following exposure to IMD_AI_. IMD_CF_ exposure caused greater harm to the midgut of adult bees, compromising essential cellular functions.

This study represents the first field investigation exploring the impact of oral exposure to IMD_CF_ and IMD_AI_ on *A. mellifera* larvae, pupae, and adult individuals. Field research involving commercial products is crucial for comprehending the effects of these compounds on pollinators. The results of this study have potential to contribute to more assertive measures in regulating and using pesticides. By enhancing the evaluation process and improving mitigation efforts, these insights can guide the implementation of target and effective strategies to tackle the challenges associated with the decline of bees.

## Data Availability

All data supporting the findings of this study are available within the article and its supplementary information files. Source data are provided in this paper. Any other relevant data or reagents are available from the corresponding authors upon reasonable request.
